# Enolase 1 (ENO1) and protein disulfide-isomerase associated 3 (PDIA3) regulate Wnt/β-catenin-driven trans-differentiation of murine alveolar epithelial cells

**DOI:** 10.1242/dmm.019117

**Published:** 2015-08-01

**Authors:** Kathrin Mutze, Sarah Vierkotten, Jadranka Milosevic, Oliver Eickelberg, Melanie Königshoff

**Affiliations:** 1 Comprehensive Pneumology Center (CPC), Helmholtz Zentrum München, University Hospital, Ludwig-Maximilians University, 81377 Munich, Member of the German Center for Lung Research (DZL), Germany; 2 University of Pittsburgh Medical Center, Pittsburgh, PA 15213-258, USA

**Keywords:** Alveolar epithelial cells, Differentiation, Lung injury and repair, Beta-catenin, Wnt pathway, Fibrosis

## Abstract

The alveolar epithelium represents a major site of tissue destruction during lung injury. It consists of alveolar epithelial type I (ATI) and type II (ATII) cells. ATII cells are capable of self-renewal and exert progenitor function for ATI cells upon alveolar epithelial injury. Cell differentiation pathways enabling this plasticity and allowing for proper repair, however, are poorly understood. Here, we applied proteomics, expression analysis and functional studies in primary murine ATII cells to identify proteins and molecular mechanisms involved in alveolar epithelial plasticity. Mass spectrometry of cultured ATII cells revealed a reduction of carbonyl reductase 2 (CBR2) and an increase in enolase 1 (ENO1) and protein disulfide-isomerase associated 3 (PDIA3) protein expression during ATII-to-ATI cell trans-differentiation. This was accompanied by increased Wnt/β-catenin signaling, as analyzed by qRT-PCR and immunoblotting. Notably, ENO1 and PDIA3, along with T1α (podoplanin; an ATI cell marker), exhibited decreased protein expression upon pharmacological and molecular Wnt/β-catenin inhibition in cultured ATII cells, whereas CBR2 levels were stabilized. Moreover, we analyzed primary ATII cells from mice with bleomycin-induced lung injury, a model exhibiting activated Wnt/β-catenin signaling *in vivo*. We observed reduced CBR2 significantly correlating with surfactant protein C (SFTPC), whereas ENO1 and PDIA3 along with T1α were increased in injured ATII cells. Finally, siRNA-mediated knockdown of ENO1, as well as PDIA3, in primary ATII cells led to reduced T1α expression, indicating diminished cell trans-differentiation. Our data thus identified proteins involved in ATII-to-ATI cell trans-differentiation and suggest a Wnt/β-catenin-driven functional role of ENO1 and PDIA3 in alveolar epithelial cell plasticity in lung injury and repair.

## Abstract (precis)

**Summary:** The authors identified proteins involved in Wnt/β-catenin-driven alveolar epithelial plasticity in lung injury and repair.

## INTRODUCTION

Chronic lung diseases, such as chronic obstructive pulmonary disease (COPD) and idiopathic pulmonary fibrosis (IPF), represent a significant health burden worldwide owing to their progressive nature and the current lack of curative treatments. Therapeutic options improving disease pathology or attenuating disease progression are limited (Barnes, 2014; Fernandez and Eickelberg, 2012).

The alveolar epithelium constitutes a major site of injury and tissue destruction in chronic lung disease and thus represents an area of intensive research (Tsuji et al., 2006; Camelo et al., 2014; Selman and Pardo, 2006). In the adult lung, the alveolar epithelium consists largely of two morphologically distinct epithelial cell types, which are crucial to maintain lung homeostasis. Alveolar epithelial type I (ATI) cells are elongated squamous cells with a large cell surface, which, owing to their close proximity to endothelial cells of the alveolar capillaries, facilitate gas exchange in the lung. Furthermore, ATI cells are highly water permeable, enabling and facilitating ion transport and maintenance of lung fluid balance (Dobbs et al., 2010; Johnson et al., 2002). Although ATI cells are not the most abundant cell type, they cover the largest surface area of the distal lung (Stone et al., 1992; Weibel, 2015). Alveolar epithelial type II (ATII) cells, which exhibit a cuboidal cell morphology, account for a much larger number of cells in the distal lung while covering a significant lower surface area (Stone et al., 1992). ATII cells are involved in ion transport and liquid homeostasis (Fehrenbach, 2001) but, most importantly, ATII cells are responsible for the production, storage, secretion and recycling of pulmonary surfactant, a complex mixture of lipids and proteins, lining the alveolar epithelium. Surfactant lowers the surface tension at the tissue-air barrier to allow proper inflation and deflation of the lung during breathing (Halliday, 2008; Lhert et al., 2007). Pulmonary surfactant also contributes to host defense in the lung (Strunk et al., 1988). Although the general steady-state cellular turnover of the adult lung is rather low in comparison to other organs (Hogan et al., 2014), recent studies have demonstrated repair capacity of the lung in response to injury (Butler et al., 2012). In a variety of different lung injury models, such as bleomycin-induced fibrosis, hyperoxia or viral infection, ATII cells have been described to serve as progenitor cells for ATI cells (Rock et al., 2011; Desai et al., 2014; Liu et al., 2011). Furthermore, recent studies utilizing linage tracing technology have established that ATII cells are capable of long-term self-renewal, indicating that these cells represent a major stem-cell population in the adult alveolar epithelium (Barkauskas et al., 2013; Desai et al., 2014).

Pathways enabling the activation and plasticity of this cell population in response to injury and allowing for proper repair, however, are poorly understood. Established markers to define ATII and ATI cell phenotypes do exist, such as surfactant proteins and T1α (podoplanin), respectively. Markers that accurately reflect the differentiation status of alveolar epithelial cells especially during injury and repair processes, however, are not well characterized. Here, we applied the model of ATII cell cultivation *in vitro*, in which ATII cells trans-differentiate into an ATI-cell-like phenotype during primary culture (Borok et al., 1994; DeMaio et al., 2009; Bhaskaran et al., 2007), to mimic differentiation and repair processes. Recent studies have reported that the Wnt/β-catenin pathway, an essential developmental pathway, is activated during alveolar epithelial cell injury and repair in general (Selman et al., 2008; Königshoff et al., 2009; Beers and Morrisey, 2011; Ulsamer et al., 2012; Tanjore et al., 2013), and in ATII-to-ATI cell trans-differentiation in particular (Flozak et al., 2010; Marconett et al., 2013). The detailed mechanism, however, of how Wnt/β-catenin signaling mediates its cellular effects on ATII-to-ATI cell trans-differentiation, remains elusive.

**TRANSLATIONAL IMPACT**

**Clinical issue**

Chronic lung diseases, such as chronic obstructive pulmonary disease (COPD) and idiopathic pulmonary fibrosis (IPF), represent a major health burden worldwide, with no curative treatment options currently available. These disease entities are characterized by reduced repair capacity of the alveolar compartment, in particular by impaired trans-differentiation of alveolar epithelial type II (ATII) to alveolar type I (ATI) cells. Thus, advances in our understanding of alveolar epithelial plasticity during lung injury and repair are of utmost importance. The characterization of relevant *in vitro* systems is required to underpin their validity and suitability for mechanistic studies and for identifying targets for future clinical intervention in human chronic lung diseases. In this study, the authors aimed to identify proteins involved in alveolar epithelial cell injury and repair processes.

**Results**

Using a proteomic approach, the authors reported for the first time carbonyl reductase 2 (CBR2), enolase 1 (ENO1) and protein disulfide isomerase associated 3 (PDIA3) as functional alveolar epithelial cell proteins. These proteins are altered during ATII-to-ATI cell trans-differentiation *in vitro*. Reduced CBR2 expression was accompanied by reduced expression of pro surfactant protein C (proSFTPC; an ATII cell marker). Moreover, ENO1 and PDIA3 were increased along with the ATI cell marker T1α. Notably, expression of ENO1, PDIA3 and T1α decreased upon inhibition of Wnt/β-catenin signaling (a pathway that is involved in impaired alveolar epithelial cell repair *in vitro* and *in vivo* and is suggested as a potential therapeutic target for pulmonary fibrosis) during ATII-to-ATI trans-differentiation, whereas CBR2 levels were stabilized. Moreover, in primary ATII cells from bleomycin-induced lung injury – a model exhibiting activated Wnt/β-catenin signaling and pulmonary fibrosis *in vivo* – CBR2 expression was reduced, significantly correlating with reduced pro-SFTPC, whereas ENO1, PDIA3 and T1α were increased. Finally, loss of ENO1 and PDIA3 function in primary ATII cells led to reduced T1α expression, indicating their functional role in alveolar epithelial cell plasticity.

**Implications and future directions**

In summary, these data validate the ATII-to-ATI cell trans-differentiation *in vitro* system as a suitable model of alveolar epithelial cell injury and wound repair *in vivo*. In addition, this study implies CBR2, ENO1 and PDIA3 as newly identified alveolar epithelial cell proteins involved in β-catenin-driven alveolar epithelial cell plasticity. Therefore, these proteins might represent potential drug targets in chronic lung disease.

The aim of the presented study was to identify proteins controlling ATII-to-ATI cell trans-differentiation using expression analysis, 2D gel electrophoresis and mass spectrometry of cultured primary murine ATII cells *in vitro*. Secondly, we sought to define molecular programs and markers for differentiation associated with the Wnt/β-catenin pathway and to investigate their relevance for injury and repair processes in the alveolar lung epithelium. We present for the first time evidence for β-catenin-dependent ENO1 and PDIA3 expression in alveolar epithelial injury and repair processes.

## RESULTS

### Dynamic changes of alveolar epithelial markers during ATII-to-ATI cell trans-differentiation

**Fig. 1.**
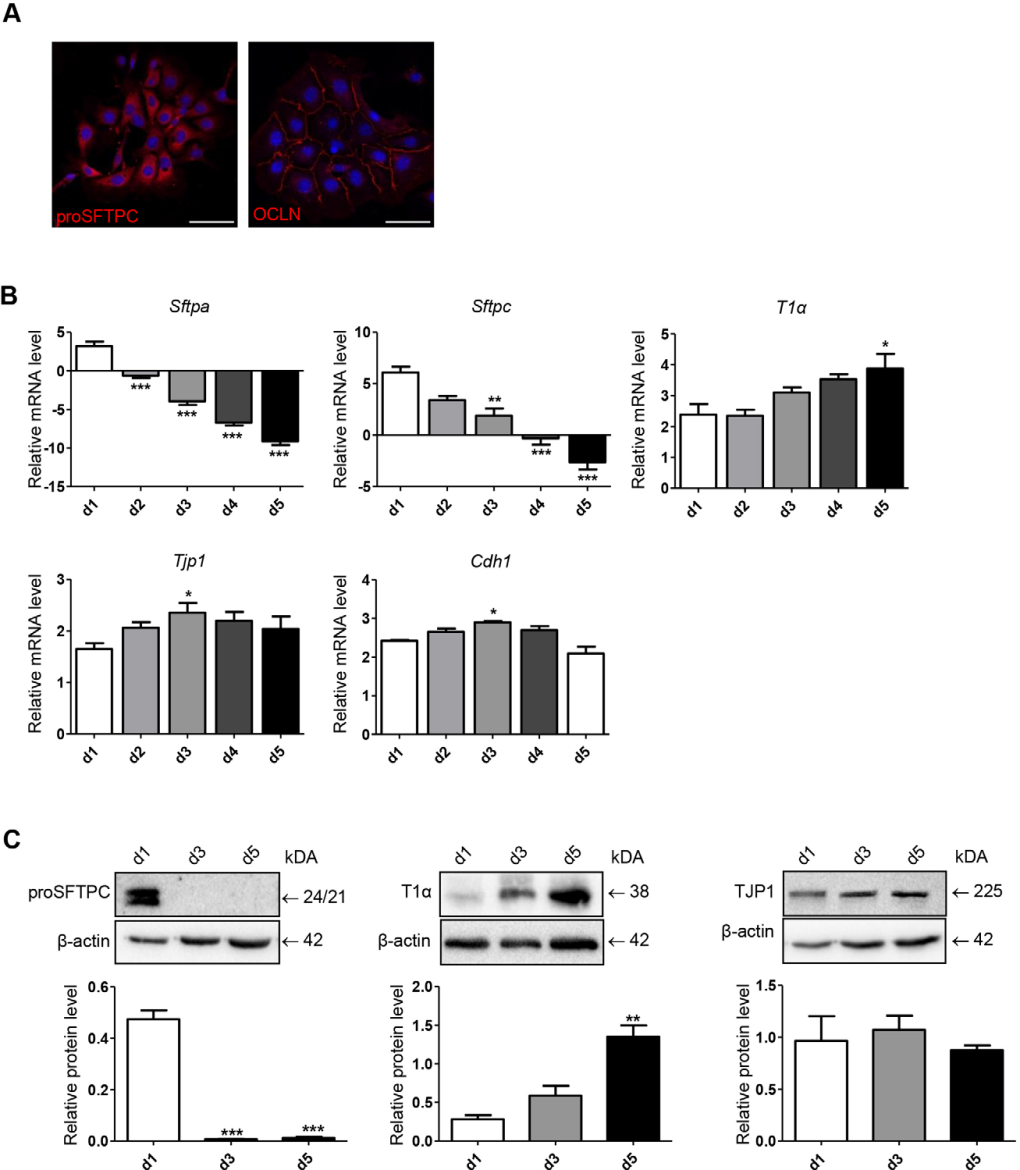
**Dynamic mRNA and protein expression changes during ATII-to-ATI cell trans-differentiation *in vitro*.** (A) Immunofluorescence staining of pmATII cells on coverslips for epithelial cell marker expression at day 2 after isolation. Fluorescent images represent a 400× magnification. The scale bar represents 50 µm. (B) mRNA expressions of epithelial cell markers during the culture of pmATII cells over a period of 5 days. mRNA levels were measured by quantitative RT-PCR (qRT-PCR) and normalized to *Hprt* as housekeeping gene. Data represent means of ΔCt values+s.e.m. of at least three independent experiments. (C) Protein expression of epithelial markers in cultured pmATII cells. Cells were lysed at the indicated time points and 15 µg of total protein per sample was subjected to immunoblot analysis. β-actin expression served as loading control. A representative experiment and a densitometric analysis of at least three independent experiments are shown. Means at indicated time points were compared to day 1 (d1) using one-way ANOVA, followed by Dunnett's post-hoc test. Significance: *P<0.05; **P<0.01; ***P<0.001.

Isolated primary murine (pm) ATII cells exhibited a robustly high purity and viability, and expressed specific ATII cell-marker and tight-junction proteins as previously published (Königshoff et al., 2009; Aumiller et al., 2013). Cytoplasmic expression of the ATII cell marker pro surfactant protein C (proSFTPC) and the tight-junction protein occludin (OCLN) is shown in Fig. 1A. The pmATII cells were cultured on cell culture dishes over a period of 5 days to induce ATII-to-ATI cell trans-differentiation and were further analyzed for their expression pattern of ATII cell (*Sftpc* and *Sftpa*) and ATI cell (*T1α*) markers, as well as for tight-junction proteins. As displayed in Fig. 1, ATII cells are characterized by a high mRNA and protein expression level of ATII cell markers *Sftpc* and *Sftpa*, which decreased significantly over the culture period. By contrast, whereas we observed rather stable expression of tight-junction proteins, the expression of the ATI cell marker *T1α* [podoplanin (*Pdpn*)] significantly increased over time, accompanied by a more flattened cell morphology (Fig. 1B,C). Analysis of additional markers involved in ATII-to-ATI cell trans-differentiation, such as forkhead box M1 (*FoxM1*), a transcription factor described to be essential for ATII-to-ATI cell trans-differentiation in an influenza lung injury model (Liu et al., 2011), as well as advanced glycosylation end product-specific receptor (*Ager*), further supported a phenotypical switch at day 3 of culture (supplementary material Fig. S1). These data demonstrate a differentiation of pmATII cells towards an ATI-cell-like phenotype under the applied culture conditions.

### Proteomic analysis of trans-differentiating alveolar epithelial cells revealed CBR2, ENO1 and PDIA3 to be differentially expressed

**Fig. 2.**
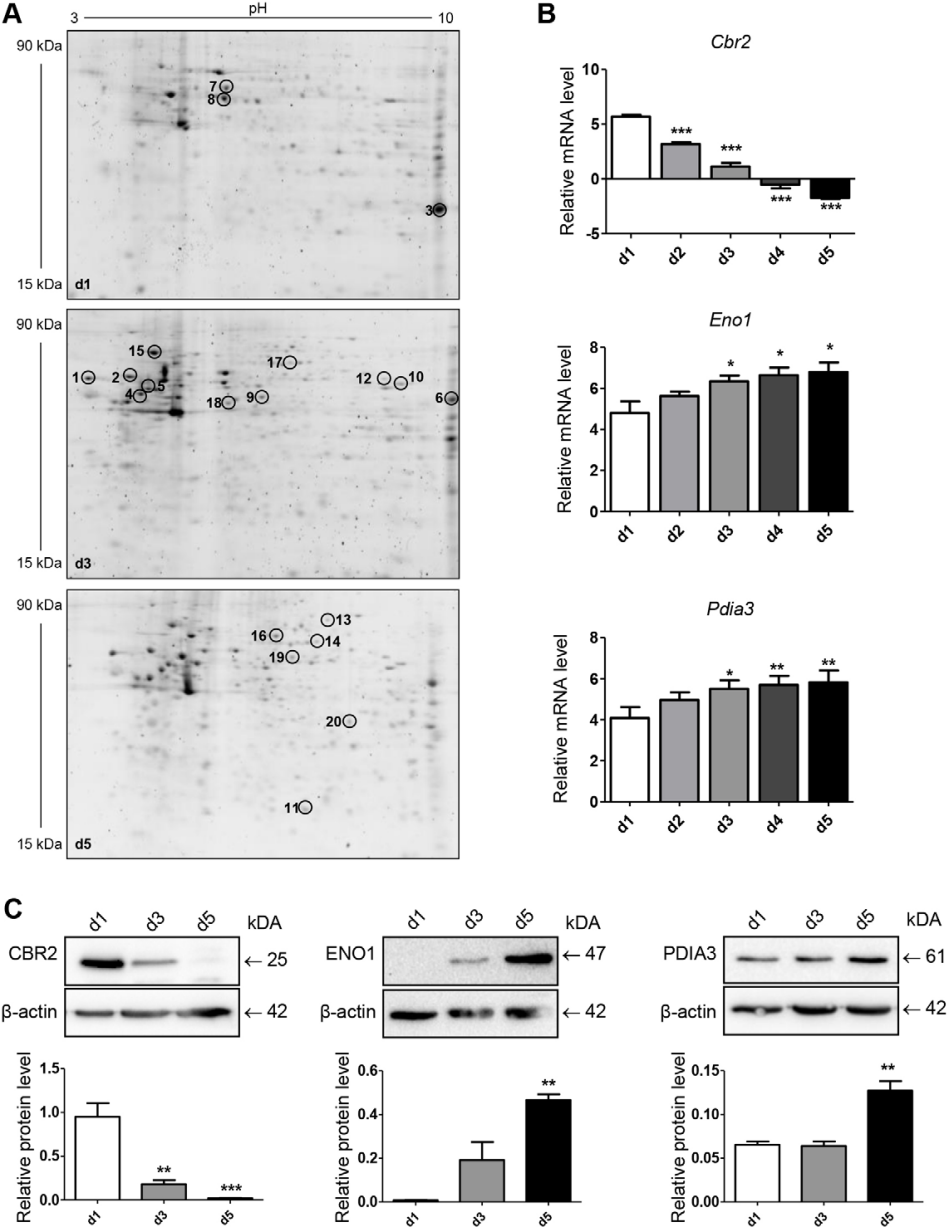
**CBR2, ENO1 and PDIA3 are differentially expressed during ATII-to-ATI cell trans-differentiation.** pmATII cells were isolated and cultured for the indicated time points (days). (A) Protein profile of primary mouse alveolar epithelial cells cultured for 1, 3 or 5 days was generated by subjecting 30 µg total protein to a 2D-PAGE. Circles mark protein spots which were identified and quantified by MALDI-TOF mass spectrometry and are differently expressed at the indicated time points. A representative image of three independent experiments for each time point is shown. (B) mRNA expression of *Cbr2*, *Eno1* and *Pdia3* was determined by qRT-PCR and normalized to *Hprt*. Data represent means of ΔCt values+s.e.m. of at least three independent experiments. (C) Protein expression was determined by subjecting 15 µg of total protein per sample to immunoblot analysis. β-actin expression served as loading control. A representative experiment and a densitometric analysis of at least three independent experiments are shown. Means at indicated time points were compared to day 1 (d1) using one-way ANOVA, followed by the Dunnett's post-hoc test. Significance: *P<0.05; **P<0.01; ***P<0.001.

To identify proteins involved in alveolar epithelial differentiation and wound repair, we applied a proteomics approach by using 2D gel electrophoresis (2DE) and subsequent mass spectrometry (MS) analysis of pmATII cells cultured for 1, 3 or 5 days. A representative image of 2D gels at day 1, day 3 and day 5 is shown in Fig. 2A. The analysis revealed several proteins to be differentially expressed in this process (Table 1 and supplementary material Table S1). We verified and confirmed differentially expressed proteins identified by 2DE and MS, using qPCR and immunoblotting. Most proteins, including carbonyl reductase 2 (CBR2), enolase 1 (ENO1; also known as α-enolase) and protein disulfide-isomerase associated 3 (PDIA3), were differentially expressed at the mRNA level and protein level over time of culture (Fig. 2B,C).

**Table 1.**
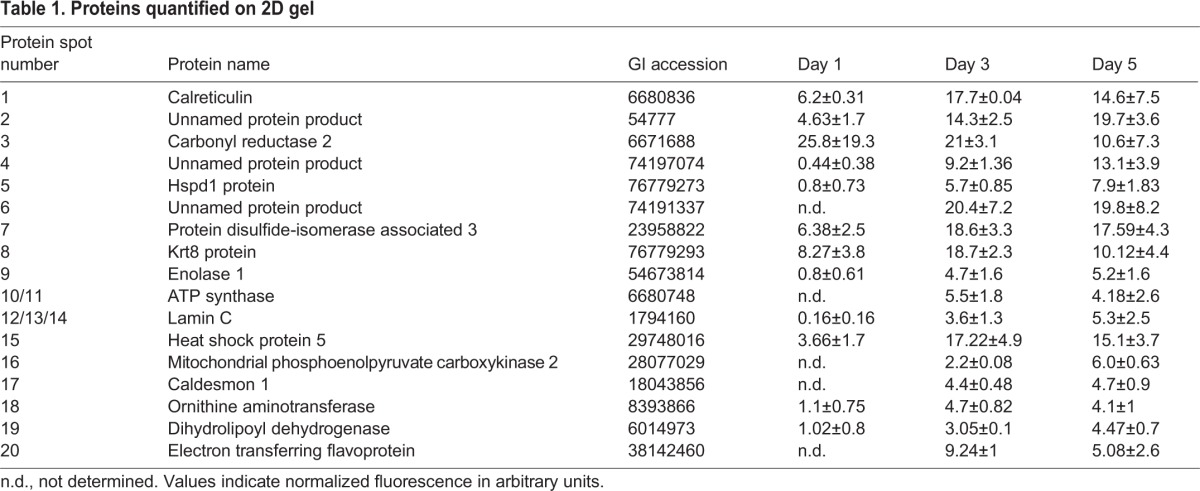
**Proteins quantified on 2D gel**

Interestingly, CBR2, also known as ‘mouse lung carbonyl reductase’ (MLCR) was one of the most downregulated proteins over the culture period, with very similar kinetics compared to proSFTPC expression. Moreover, ENO1, a protein involved in glycolytic processes, and PDIA3 [also named ERp57 and GRP58 (Turano et al., 2011)], a protein disulfide isomerase, were significantly induced and PDIA3 protein localized to T1α-positive cultured alveolar epithelial cells at day 3 of culture (Fig. 2C and supplementary material Fig. S2). The described role of CBR2 and ENO1 in cellular differentiation in other organs (Wenz et al., 1992; Lopez-Alemany et al., 2003; Ryu et al., 2012) prompted us to further investigate the relevance of these proteins in the context of alveolar epithelial differentiation. Furthermore, PDIA3 is involved in the quality control of newly synthesized glycoproteins, suggesting a possible connection to aberrantly activated Wnt/β-catenin signaling in lung injury and repair (Chilosi et al., 2003; Königshoff et al., 2008, 2009).

### Alveolar epithelial cell trans-differentiation is accompanied by an activation of the Wnt/β-catenin pathway

**Fig. 3.**
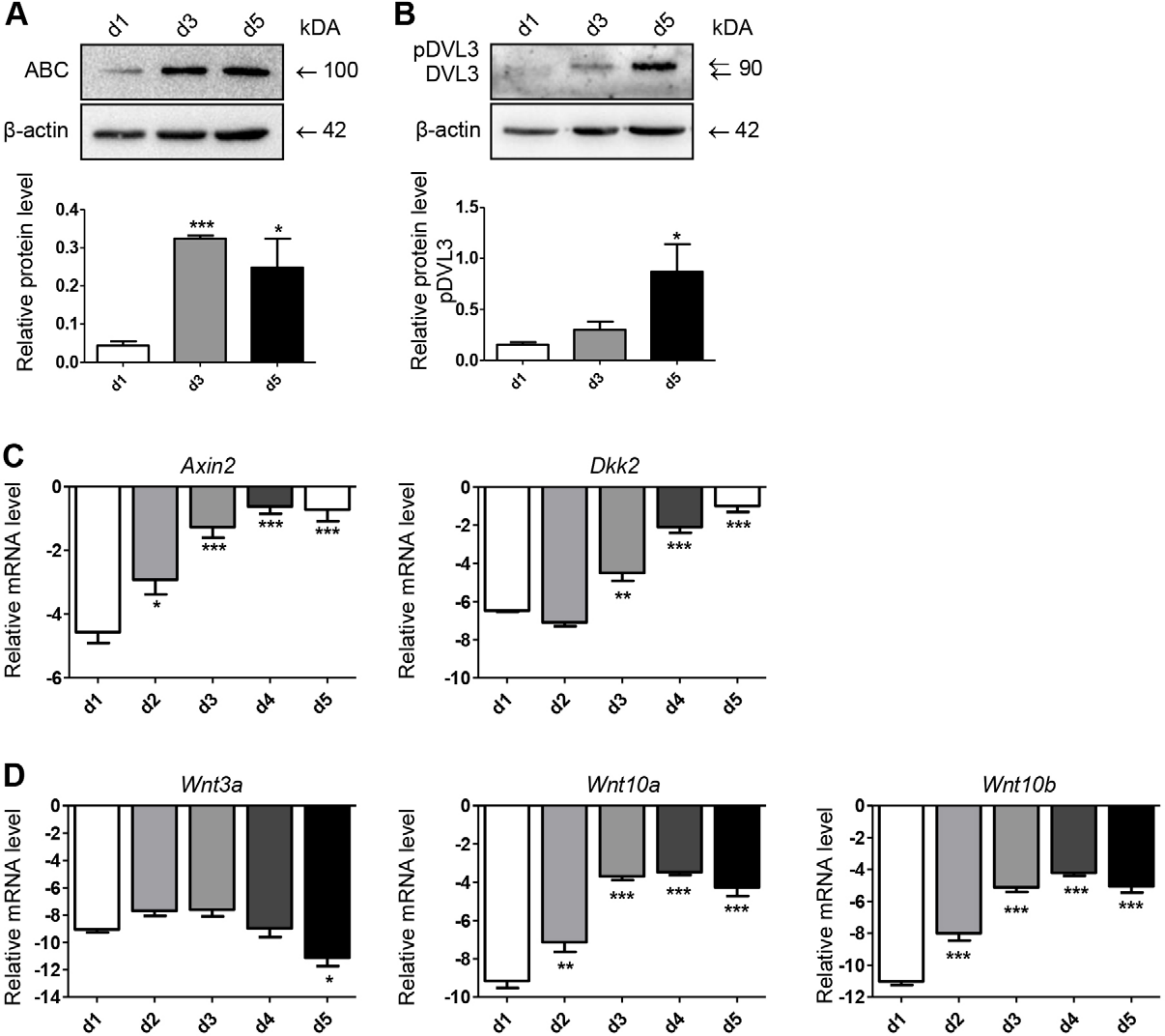
**Wnt/β-catenin pathway is activated during ATII-to-ATI cell trans-differentiation.** pmATII cells were isolated and cultured for the indicated time points (days). (A,B) Cells were lysed using T-Per lysis buffer containing protease inhibitors and 15 µg of total protein per sample was subjected to immunoblot analysis. β-actin expression served as loading control. A representative experiment and a densitometric analysis of at least three independent experiments is shown. ABC, active β-catenin. (C) mRNA expression of Wnt/β-catenin target genes. (D) mRNA expression of canonical Wnt ligands. mRNA expression was measured by qRT-PCR and normalized to *Hprt*. Data represent means of ΔCt values+s.e.m. of at least three independent experiments. Means at indicated time points were compared to day 1 (d1) using one-way ANOVA, followed by the Dunnett's post-hoc test. Significance: *P<0.05; **P<0.01; ***P<0.001.

Because the Wnt/β-catenin pathway has been described to be involved in relevant developmental and regenerative processes in the lung in general (Beers and Morrisey, 2011), as well as in lung injury and repair processes in particular (Flozak et al., 2010; Aumiller et al., 2013; Königshoff et al., 2009), we asked the question, are the newly identified proteins linked to Wnt/β-catenin signaling? First, we investigated Wnt/β-catenin activity in our model. Importantly, we found a considerably increased level of active β-catenin (ABC) at day 3 and day 5 accompanied by increased T1α expression (Figs 3A and 1C), which is in accordance to previous findings in rat alveolar epithelial cells (Flozak et al., 2010). In order to determine whether the activation of β-catenin is mediated by a Wnt-ligand-dependent signaling process, we determined the expression and phosphorylation status of dishevelled segment polarity protein 3 (DVL3), a cytoplasmic protein, which is phosphorylated and therefore activated upon binding of a Wnt ligand to a Wnt receptor (frizzled) and co-receptor (low-density lipoprotein receptor-related protein 5 or 6). As displayed in Fig. 3B, we observed an increase in DVL3 phosphorylation as early as day 3, indicating that activation of β-catenin is driven at least in part by a Wnt-ligand/receptor-dependent mechanism. This was further substantiated by a significant increase of mRNAs of the direct β-catenin-dependent target genes *Axin2* and Dickkopf-related protein 2 (*Dkk2*), detected during the trans-differentiation of ATII cells [Fig. 3C; *Axin2*: ΔCt day 1 (d1) −4.56+0.34 s.e.m., ΔCt day 5 (d5) −0.72+0.37 s.e.m., *P*<0.001; *Dkk2*: ΔCt d1 −6.47+0.06 s.e.m., ΔCt d5 −0.99+0.31 s.e.m., *P*<0.001].

Next, we investigated the expression of the canonical Wnt ligands *Wnt3a*, *Wnt10a* and *Wnt10b* (Baarsma et al., 2013) to further clarify which Wnt ligands might induce active Wnt signaling in this process. Notably, we found that *Wnt10a* and *Wnt10b*, but not *Wnt3a*, exhibited a remarkable increased expression as early as day 2, linking these Wnt ligands to ATII-to-ATI cell trans-differentiation (Fig. 3D; *Wnt10a*: ΔCt d1 −9.15+0.63 s.e.m., ΔCt d5 −4.27+0.79 s.e.m., *P*<0.001; *Wnt10b*: ΔCt d1 −11.01+0.40 s.e.m., ΔCt d5 −5.05+0.67 s.e.m., *P*<0.001).

### Inhibition of β-catenin activation inhibits pmATII cell trans-differentiation and regulates CBR2, ENO1 and PDIA3 expression

**Fig. 4.**
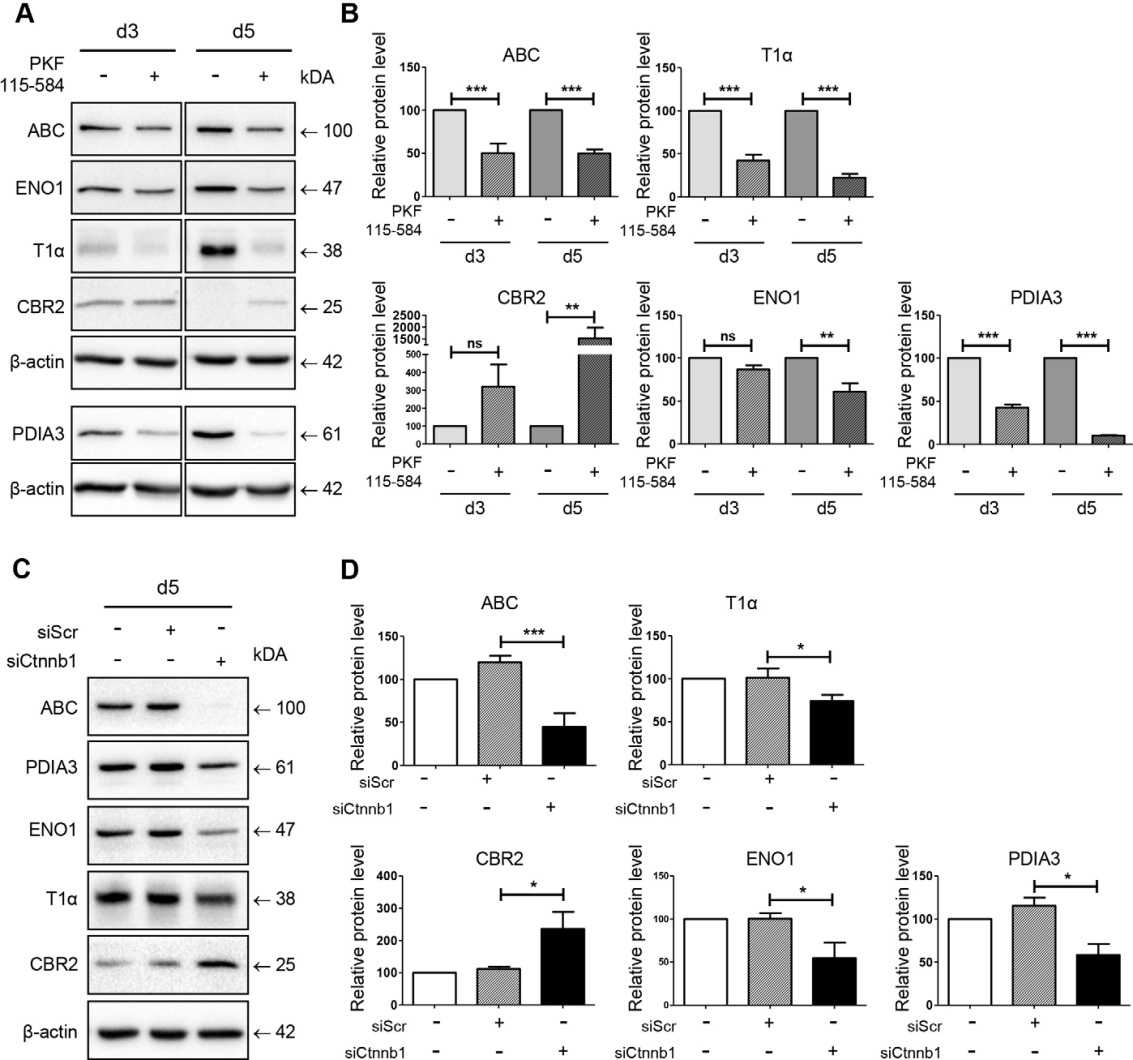
**β-catenin inhibition alters ATII-to-ATI cell trans-differentiation along with CBR2, ENO1 and PDIA3 expression.** (A) pmATII were treated with PKF115-584 (1 µM) or DMSO as control at day 1 after isolation until day 3 and day 5, respectively. Treated cells were lysed and subjected to immunoblot analysis. β-actin expression served as loading control. A representative experiment is shown. (B) Densitometric analysis of at least three independent experiments using PKF115-584 treatment. Means of the indicated groups were compared to time-matched treatment controls using one-way ANOVA, followed by Bonferroni multiple comparison test. Significance: **P<0.01; ***P<0.001; ns, not significant. (C) pmATII cells were transfected using an siRNA pool targeting *Ctnnb1* and a scrambled (siScr) control sequence, respectively. Non-transfected cells served as additional control. At day 5 cells were lysed and subjected to immunoblot analysis. A representative experiment is shown. (D) Quantification of at least three independent experiments of *Ctnnb1* siRNA treatments. Means were compared to time-matched transfection control (siScr), using one-way ANOVA, followed by Bonferroni multiple-comparison test. Significance: *P<0.05; ***P<0.001.

The activation of the Wnt/β-catenin pathway in our model of ATII-to-ATI cell trans-differentiation prompted us to evaluate if the newly identified proteins are regulated by active β-catenin signaling. In order to do so, we treated pmATII cells with PKF115-584, a well-known compound inhibiting the T cell factor (TCF)–β-catenin complex (Lepourcelet et al., 2004). Inhibition of β-catenin in ATII cells led to significantly decreased expression of ABC at day 3 and day 5, indicating successful inhibition in pmATII cells (Fig. 4A,B). Moreover, the inhibition of ABC led to a significant inhibition of T1α induction at day 3 (d3) and day 5 (d5), and thus blocked the development of an ATI-like phenotype (Fig. 4A,B; d3 42+6.83 s.e.m. % of control, *P*<0.001; d5 22+4.84 s.e.m. % of control, *P*<0.001). Notably, determining the influence of β-catenin inhibition on the markers identified by the proteomic approach revealed a significantly reduced induction of ENO1 (d3 86.00+5.07 s.e.m. % of control, *P*=ns; d5 60.88+9.89 s.e.m. % of control, *P*<0.01) and PDIA3 (d3 42.70+3.59 s.e.m. % of control, *P*<0.001; d5 10.01+0.80 s.e.m. % of control, *P*<0.001) (Fig. 4A,B). In contrast, the expression of CBR2, which was lost over time until day 5 under normal conditions, was still detectable at day 5 and therefore significantly stabilized (d3 321.5+123.1 s.e.m. % of control, *P*=ns; d5 1521+453.4 s.e.m. % of control, *P*<0.01) (Fig. 4A,B). We confirmed our results by using another β-catenin inhibitor, which has already been applied in an experimental lung fibrosis model *in vivo* (ICG-001) (Henderson et al., 2010) (supplementary material Fig. S3). Furthermore, we utilized an independent approach to inhibit β-catenin signaling using siRNA-mediated downregulation of *Ctnnb1* (β-catenin). Importantly, β-catenin knockdown also led to decreased expression of the ATI marker T1α as well as reduced ENO1 and PDIA3 expression in cultured AT cells, whereas CBR2 expression was restored, thus further corroborating the previous findings achieved by pharmacological inhibition (Fig. 4C,D). In a complementary approach, we evaluated whether further activation of Wnt/β-catenin signaling leads to enhanced trans-differentiation of pmATII cells as well as PDIA3 and ENO1 expression. To this end, we applied the glycogen synthase kinase-3 (GSK3) inhibitor CT99021, which is a well-known activator of β-catenin (Uhl et al., 2015). Indeed, we observed an induction of T1α, ENO1 and PDIA3; however, this did not reach statistical significance, indicating that intrinsic activated β-catenin signaling might already have reached maximal induction (supplementary material Fig. S4).

Taken together, our data strongly support the notion that active β-catenin signaling regulates ENO1, PDIA3 and CBR2 protein expression in alveolar epithelial cells *in vitro*.

### Bleomycin-induced lung injury regulates CBR2, ENO1 and PDIA3 levels in pmATII cells

**Fig. 5.**
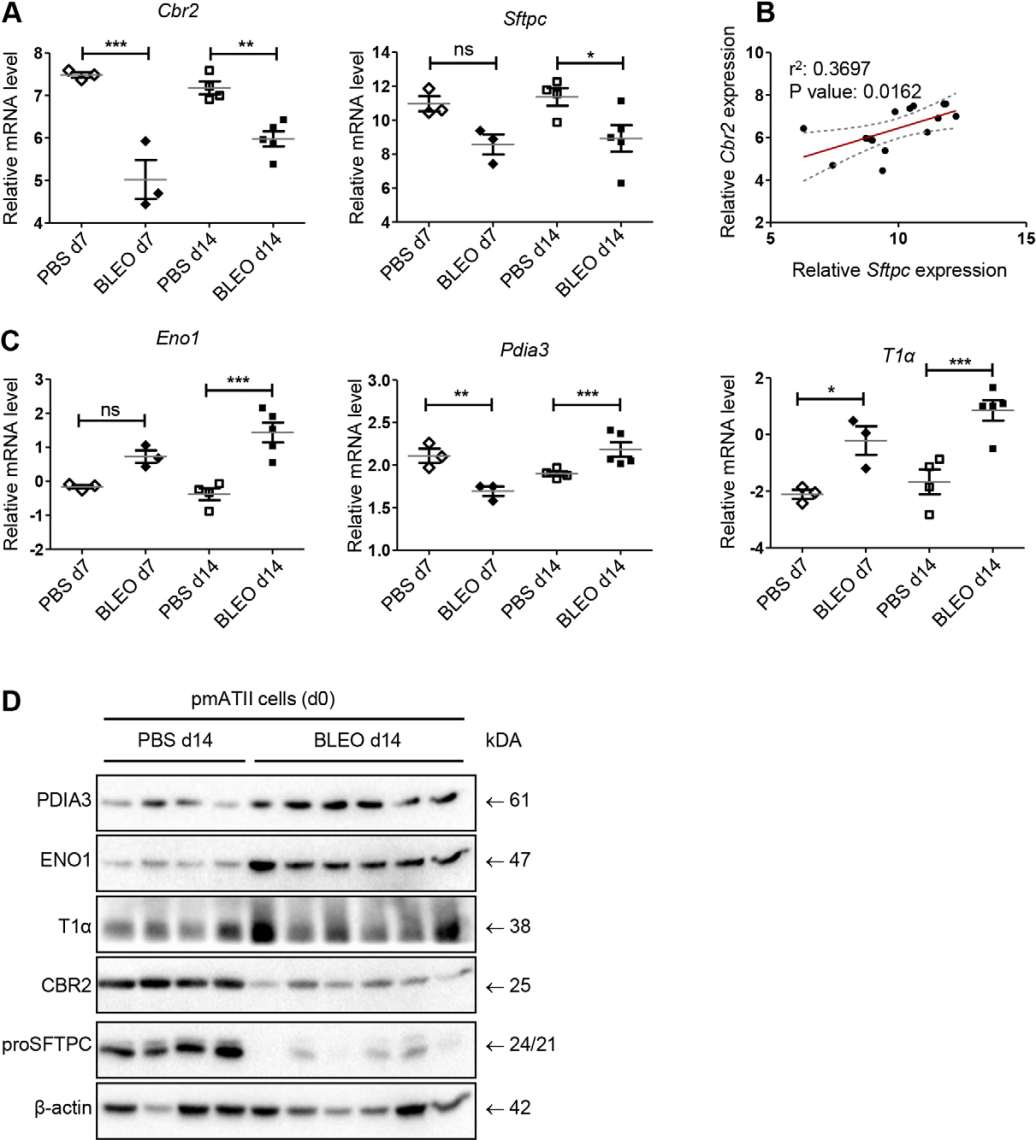
**ENO1, PDIA3 and CBR2 expression is altered in injured pmATII cells.** Mice were instilled with either PBS or bleomycin (BLEO) (5 U/kg body weight). At day 7 or day 14 after instillation, mice were sacrificed and lungs of four PBS- and four bleomycin-treated mice were pooled for pmATII cell isolation. (A) Freshly isolated pmATII cells (day 0) were analyzed for mRNA expression of *Cbr2* and *Sftpc*. (B) Correlation analysis of mRNA expression of *Cbr2* and *Sftpc* using a linear regression model. Data points represent ΔCt values for the respective genes. The corresponding regression line is indicated in red. 95% confidence intervals are depicted by a gray scattered line. *r*^2^ and *P*-value is given in the graph for the compared variables. (C) *Eno1*, *Pdia3* and *T1α* mRNA expression is shown using qRT-PCR. Means of the indicated groups were compared using one-way ANOVA, followed by Bonferroni multiple-comparison test. Significance: *P<0.05; **P<0.01; ***P<0.001; ns, not significant. (D) Protein expression of the indicated proteins in freshly isolated pmATII cells from PBS-treated or BLEO-treated mice at day 14 after isolation. β-actin expression served as loading control.

Next, we aimed to investigate whether the newly identified proteins are involved in alveolar epithelial cell injury and repair processes *in vivo*. Therefore, we utilized the murine bleomycin-induced lung injury model, in which active Wnt/β-catenin signaling has been demonstrated in alveolar epithelial cells (Königshoff et al., 2009; Flozak et al., 2010). We isolated pmATII cells at day 7 and day 14 after instillation and subsequently analyzed the freshly isolated cell population (d0) on mRNA (Fig. 5A-C) and protein (Fig. 5D) level. The analysis revealed a significant reduction of *Cbr2* mRNA expression in pmATII cells derived from bleomycin-instilled mice compared to phosphate-buffered saline (PBS)-treated mice with a concurrent reduction in ATII-cell-marker *Sftpc* expression (Fig. 5A; *Cbr2*: ΔCt d7 PBS 7.48+0.06 s.e.m.; ΔCt d7 BLEO 5.02+0.46, *P*<0.001; ΔCt d14 PBS 7.18+0.15 s.e.m.; ΔCt d14 BLEO 5.98+0.18 s.e.m., *P*<0.01; *Sftpc*: ΔCt d7 PBS 10.96+0.45 s.e.m.; ΔCt d7 BLEO 8.57+0.59 s.e.m., *P*=ns; ΔCt d14 PBS 11.36+0.52 s.e.m.; ΔCt d14 BLEO 8.92+0.78 s.e.m., *P*<0.05). Importantly, combined analysis of *Cbr2* and *Sftpc* expression using a linear regression model revealed a significant correlation of the expression of both proteins (*r*^2^: 0.3697; *P*-value: 0.0162) (Fig. 5B), suggesting that similar regulatory pathways are involved in their expression. Similarly, we found increased levels of *Eno1* and *T1α* at day 7 as well as at day 14 (Fig. 5C; *Eno1*: ΔCt d14 PBS −0.38+0.17 s.e.m.; ΔCt d14 BLEO 1.43+0.29 s.e.m., *P*<0.05; *T1α*: ΔCt d14 PBS −1.67+0.43 s.e.m.; ΔCt d14 BLEO 0.85+0.35, *P*<0.001), whereas *Pdia3* levels were decreased at day 7 but increased at day 14 upon bleomycin-induced lung injury (Fig. 5C; *Pdia3*: ΔCt d7 PBS 2.11+0.08 s.e.m.; ΔCt d7 BLEO 1.69+0.06, *P*<0.01; ΔCt d14 PBS 1.9+0.03 s.e.m.; ΔCt d14 BLEO 2.18+0.08, *P*<0.001). These findings were further confirmed on protein level (Fig. 5D). We determined increased ENO1, PDIA3 and T1α protein expression accompanied by decreased CBR2 and proSFTPC protein expression in injured ATII cells isolated 14 days after bleomycin-induced lung injury compared to PBS-treated lungs (Fig. 5D). These data suggest that there is an ongoing epithelial cell wound repair attempt *in vivo* as early as 7 days after induction of injury and that this response is characterized by increased expression of ENO1, PDIA3 and T1α.

### siRNA-mediated knockdown of ENO1 and PDIA3 reduces differentiation of pmATII cells

**Fig. 6.**
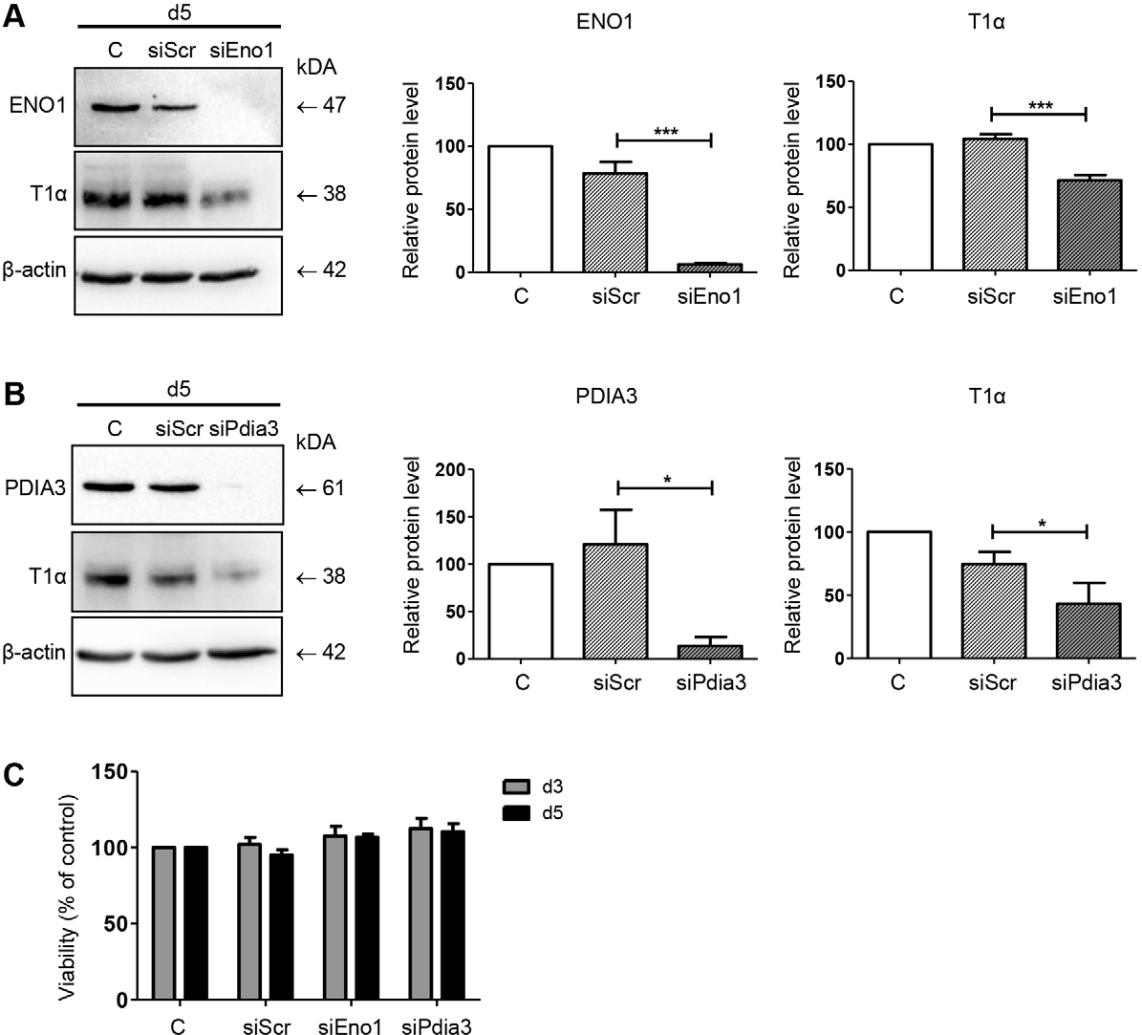
**The siRNA-mediated knockdown of ENO1 and PDIA3 inhibits ATII-to-ATI cell trans-differentiation.** pmATII cells were transfected at day 2 using an siRNA pool targeting *Eno1*, *Pdia3* and a scrambled (siScr) control sequence, respectively. Non-transfected cells served as additional control. At day 5 cells were lysed and subjected to immunoblot analysis. Knockdown efficiency at day 5 was determined by detection of ENO1 and PDIA3 protein, respectively. A representative experiment and a quantification of three independent experiments of T1α expression upon (A) ENO1 and (B) PDIA3 knockdown are shown. Means were compared to time-matched transfection control (siScr) using one-way ANOVA followed by Bonferroni multiple-comparison test. Significance: *P<0.05; ***P<0.001. (C) Viability of pmATII cells transfected with siScr, siEno1 or siPdia3, respectively. Analysis was performed at day 3 and day 5 using the WST-1 assay. Data were measured in triplicates and were normalized to untreated control cells at the respective time points.

Finally, we asked whether modulation of ENO1 or PDIA3 expression impacts the trans-differentiation process from pmATII cells towards a more ATI-cell-like phenotype. Therefore, we induced siRNA-mediated knockdown of ENO1 and PDIA3, respectively, in pmATII cells. As shown in Fig. 6A, the treatment of pmATII cells using an *ENO1*-targeting siRNA pool significantly reduced ENO1 expression until day 5 [scrambled control siRNA (siScr) 78.4+9.31 s.e.m. % of control; siEno1 6.33+0.93 s.e.m. % of control, *P*<0.001]. Notably, ENO1 knockdown significantly inhibited T1α induction compared to pmATII cells treated with the scrambled control (siScr 104.3+3.82 s.e.m. % of control; siEno1 71.39+4.19 s.e.m. % of control, *P*<0.001). Similarly, we found reduced T1α expression in pmATII cells at day 5 using an siRNA pool efficiently targeting PDIA3 (Fig. 6B; siScr 74.72.3+9.75 s.e.m. % of control; siPdia3 43.37+16.49 s.e.m. % of control, *P*<0.05). Importantly, knockdown of either ENO1 or PDIA3 did not affect cell viability in T1α-expressing cells, as shown by WST-1 analysis (Fig. 6C). Taken together, these data suggest a functional role of ENO1 and PDIA3 for the process of β-catenin-driven ATII-to-ATI cell trans-differentiation, and injury and repair, *in* *vitro* and *in vivo*.

## DISCUSSION

Alveolar epithelial injury and remodeling is strongly associated with chronic lung diseases. To date, several studies have demonstrated that the population of ATII cells possesses properties of stem cells within the adult lung alveolar epithelium *in vivo* (Adamson and Bowden, 1974; Chapman et al., 2011; Barkauskas et al., 2013; Treutlein et al., 2014; Desai et al., 2014; Vaughan et al., 2015). In-depth characterization of alveolar epithelial subpopulations and their function during homeostasis and disease is hampered by a limited number of marker proteins. Here, we aimed to identify previously unknown alveolar epithelial cell proteins and thus get deeper insight into the molecular mechanism and programs of alveolar epithelial cell differentiation, which is a prerequisite for proper repair induction. To this end, we initially utilized the model of murine ATII-to-ATI cell trans-differentiation in primary culture *in vitro*. We report, for the first time, that CBR2, ENO1 and PDIA3 are functional alveolar epithelial cell proteins. We further investigated the relevance of these proteins for alveolar epithelial cell trans-differentiation and cellular plasticity functionally *in vitro* as well as in the bleomycin-induced lung injury mouse model *in vivo*, demonstrating their involvement in alveolar epithelial injury and repair mechanisms in a β-catenin-dependent manner.

The trans-differentiation of primary ATII cells into ATI cells *in vitro* is a widely applied model to investigate alveolar epithelial cell phenotypes and function, and primary ATII cells from various species have been used (Flozak et al., 2010; Zhao et al., 2013; Ghosh et al., 2013; Marconett et al., 2013). In the study presented here, we detect a strong decrease of surfactant protein expression on mRNA level as well as on protein level, whereas levels of the ATI-cell-specific protein T1α increased and structural epithelial markers were stably expressed. Taken together, this suggests a change in epithelial characteristics in the direction of an ATI-like phenotype (Fig. 1B,C). These data, gained from murine ATII cells, are in line with studies largely using primary rat ATII cells that also demonstrated the loss of ATII-cell-specific proteins accompanied by an increase of ATI cell genes and stable expression of structural epithelial cell markers, such as cytokeratin (Flozak et al., 2010; Wang et al., 2013; DeMaio et al., 2009; Marconett et al., 2013). Here, we mainly based our ATI-cell-like definition on the expression of T1α along with typical morphological changes in the AT phenotype over culture. Although other ATI cell markers have been described, including aquaporin 5, HOP homeobox (Hopx1) or receptor for advanced glycosylation end products (RAGE), T1α has been established as a robust ATI cell marker conserved over species (Flozak et al., 2010; Zhao et al., 2013; Marconett et al., 2013; Barkauskas et al., 2013; Treutlein et al., 2014), has been associated with an ATI cell phenotype *in vivo* (Yee et al., 2006), and, most importantly, this has been confirmed in human lung tissue (Fujino et al., 2012; Ghaedi et al., 2014; Barkauskas et al., 2013; Marconett et al., 2013).

Although ATII cell plasticity and progenitor cell function is well-described *in vitro* and *in vivo*, data on ATI cell function and plasticity remain sparse. ATI cells are thought to be terminally differentiated cells (Crosby and Waters, 2010; Weibel, 2015). It has been reported, however, that ATI-like cells derived from *in vitro* culture are capable of switching and even in part reversing their phenotype (Borok et al., 1998; Gonzalez et al., 2009). Moreover, recent *in vivo* lineage-tracing data by Jain et al. demonstrate that ATI cells (labeled by Hopx1) can give rise to SFTPC-positive ATII cells after pneumonectomy *in vivo* as well as in lung organoid cultures (Jain et al., 2015). These important findings further underline that alveolar epithelial cells exhibit an extremely high and not yet fully understood degree of plasticity, in particular in response to lung injury (Hogan et al., 2014).

Employing a proteomics approach, we identified several proteins that have not yet been described in the context of ATII-to-ATI cell trans-differentiation. A reduced expression of CBR2, a murine carbonyl reductase, was detected in ATI-like cells, as well as in ATII cells isolated from mice displaying bleomycin-induced lung injury, following the same kinetics as the loss of proSFTPC. CBR2 has been previously described to be highly expressed in the murine lung, localizing to mitochondria of non-ciliated club cells and ciliated cells of the bronchioles, as well as ATII cells (Matsuura et al., 1994). Functionally, CBR2 is proposed to be involved in the metabolism of endogenous or inhaled carbonyl compounds (Ebert et al., 2015) and, interestingly, has also been described to be involved in cellular differentiation, such as adipocyte differentiation (Wenz et al., 1992). Furthermore, a function in lipid peroxidation (Ebert et al., 2015) has been discussed for CBR2, connecting this protein to lipid metabolism, one of highest enriched networks described in a comprehensive joint analysis of human and rat alveolar epithelial transdifferentiation by Marconett and colleagues (2013). This further suggests that CBR2 might be closely linked to surfactant metabolism. Moreover, reduced CBR2 expression was reported in a murine model of nickel-induced acute lung injury, strengthening the potential connection to alveolar epithelial lung injury (Wesselkamper et al., 2005). CBR2 closely correlated with proSFTPC expression and CBR2 levels were stabilized upon inhibition of β-catenin-dependent ATII-to-ATI cell trans-differentiation, strongly suggesting that CBR2 can be used as an additional ATII cell marker, as well as a functionally relevant protein in ATII cell homeostasis. Owing to the rapid loss of this marker *in vitro*, further functional cell culture studies are limited and future studies addressing the role of CBR2 in this context might benefit from novel 3D tissue culture models, mimicking the natural special environment in the alveolus as well as providing potential for cell-cell interactions.

We further demonstrated the induction of ENO1 in the context of differentiation and alveolar epithelial injury in ATII cells. ENO1 is a key glycolytic enzyme, displaying various functions in eukaryotic as well as prokaryotic cells. ENO1 has been linked to migration and invasion of glioma cells (Song et al., 2014). ENO1 has also been described to exert its migratory function as a plasminogen receptor at the cellular surface, supporting the degradation of extracellular matrix molecules by plasmin (Díaz-Ramos et al., 2012). Whether its upregulation during alveolar epithelial cell differentiation is linked to a migratory phenotype associated with wound repair still remains elusive. Remarkably, ENO1 seems to be required for muscle regeneration after injury in a murine model (Lopez-Alemany et al., 2003), linking this protein to repair, which supports our observation of impaired trans-differentiation and possibly wound repair of ATII cells in the presence of siRNA-mediated knockdown of ENO1 (Fig. 6A). Moreover, ENO1 was described as a serum biomarker in hepatic fibrosis, which provides additional evidence that this protein is crucial for organ injury and repair responses (Zhang et al., 2013). Furthermore, the co-regulation of ENO1 with T1α in the context of differentiation as well as induced injury is intriguing, because T1α is not only a marker for ATI cells in the adult lung but has also been implicated in migration and wound repair in the skin (Krishnan et al., 2013; Honma et al., 2012).

Similar to ENO1, we found increased expression of PDIA3 involved in β-catenin-driven ATII-to-ATI cell trans-differentiation. PDIA3 exerts diverse functions based on its expression in different subcellular compartments (Turano et al., 2011). PDIA3 exhibits a major role as molecular chaperone involved in the quality control process for newly synthesized glycoproteins in the endoplasmic reticulum (ER), interacting with calnexin or calreticulin (Oliver et al., 1999). Interestingly, we also found increased calreticulin in our *in vitro* screen as well as in injured ATII cells *in vivo* (data not shown), which might suggest that both proteins are involved in cellular stress responses *in vitro* and *in vivo*. PDIA3 has previously been associated with ER stress following lung injury (Robertson et al., 2002), as well as enhanced apoptosis and dysregulated repair (Anathy et al., 2012). In renal fibrosis, PDIA3 has been shown to contribute to extracellular matrix accumulation (Dihazi et al., 2013), which supports our observation of increased PDIA3 expression in injured and fibrotic ATII cells from bleomycin-treated lungs 14 days after injury. Taken together, these data suggest that induction of PDIA3 during ATII-to-ATI cell trans-differentiation reflects an initial (impaired) repair response, which might ultimately result in fibrosis development.

The Wnt/β-catenin pathway represents a crucial component of the attempted repair response in lung injury and fibrosis. Active signaling of developmental pathways, including the Wnt/β-catenin pathway, has been demonstrated to be involved in lung tissue development (Beers and Morrisey, 2011) and in repair and regenerative processes (Königshoff and Eickelberg, 2010; Liu et al., 2011). In particular, Wnt/β-catenin signaling has been linked to insufficient or dysregulated repair in chronic lung disease, including IPF and COPD, as well as experimental models thereof (Selman et al., 2008; Kneidinger et al., 2011; Königshoff et al., 2009; Wang et al., 2011; Uhl et al., 2015). Here, we demonstrate activation of Wnt-ligand-dependent β-catenin signaling by increased ABC and phosphorylated DVL3 levels, as well as target gene induction, implying a Wnt-ligand-dependent mechanism (Fig. 3). These results are in agreement with findings in rat ATII by Flozak et al. (2010). Here, we report that the canonical Wnt ligands *Wnt10a* and *Wnt10b* represent potential Wnt ligands driving this process and future studies will clarify the contribution of different Wnt ligands. Interestingly, overexpression of Wnt10b has been shown to contribute to the development of skin fibrosis (Akhmetshina et al., 2012). Furthermore, Wang and colleagues link the activation of Wnt/β-catenin signaling to a decreased expression of microRNA miR-375, regulating the expression of Wnt receptor frizzled 8 (FZD8), a predicted and confirmed target of miR-375 (Wang et al., 2013). Wnt/β-catenin signaling has been further identified and confirmed by a comprehensive transcriptomic and epigenomic analysis describing the Wnt/β-catenin pathway as concomitantly regulated in rat and human trans-differentiating ATII cells (Marconett et al., 2013).

Notably, expression of the newly identified proteins CBR2, ENO1 and PDIA3 was altered upon pharmacological as well as molecular β-catenin inhibition (Fig. 4, supplementary material Fig. S3), suggesting that the newly identified proteins function downstream of β-catenin signaling and thus further corroborating that β-catenin is a key regulator of alveolar epithelial cellular plasticity. Of note, we observed stabilized CBR2 level upon β-catenin inhibition, a marker that is closely linked to SFTPC expression, thereby supporting the notion that β-catenin inhibition might also promote ATII cell homeostasis and function. Protein levels of SFTPC, however, were not detectable in our cultured cells, which might be due to a limited sensitivity of the protein analysis by western blotting. Moreover, other essential factors, such as stretch and 3D structure, are missing in our culture model. Future studies using advanced ATII-to-ATI cell trans-differentiation models are needed to further investigate SFTPC metabolism upon β-catenin, ENO1 and/or PDIA3 modulation.

It has to be pointed out that the involvement of additional developmental pathways, such as transforming growth factor β (TGFβ) and bone morphogenetic protein (BMP) signaling, in alveolar epithelial ATII-to-ATI cell trans-differentiation (Bhaskaran et al., 2007; Zhao et al., 2013) has been demonstrated. This suggests a high complexity of interacting pathways with potential direct or indirect crosstalk at multiple levels. Along this line, inhibition of β-catenin signaling has been shown by several groups to attenuate bleomycin-induced lung fibrosis *in vivo* (Ulsamer et al., 2012; Henderson et al., 2010). Thus, although initial β-catenin signaling seems to be relevant to initiate wound repair, crosstalk of Wnt/β-catenin with other pathways might interfere with proper repair and result in fibrosis (Königshoff and Eickelberg, 2010; Ulsamer et al., 2012; Tanjore et al., 2013; Lam et al., 2014). Future studies are needed to further delineate signaling crosstalk, which also needs to be considered for the development of novel therapies and drugs aiming to modulate impaired lung injury and repair.

In summary, our data indicate that the model of ATII-to-ATI cell trans-differentiation *in vitro* is a suitable alveolar epithelial cell injury and wound repair model that closely mimics the ongoing repair attempts as observed in lung injury and fibrosis *in vivo*. We demonstrate that CBR2, ENO1 and PDIA3 are newly identified alveolar epithelial cell proteins involved in β-catenin-driven ATII-to-ATI cell trans-differentiation, thus contributing to alveolar epithelial cell plasticity in lung injury and fibrosis.

## MATERIALS AND METHODS

### Animals

Eight- to ten-week-old, pathogen-free female C57BL/6N mice (Charles River Laboratories, Sulzfeld, Germany) were used for all experiments, which were conducted according to the Ethics Committee guidelines of the Helmholtz Zentrum München and Government of Bavaria. Mice had free access to water and rodent laboratory chow. For the induction of lung injury, mice were subjected to intratracheal bleomycin instillation. Bleomycin sulfate (Almirall, Barcelona, Spain) was dissolved in sterile PBS and applied using the Micro-Sprayer Aerosolizer, Model IA-1C (Penn-Century, Wyndmoor, PA), as a single dose of 0.08 mg in 200 µl solution per animal (5 U/kg body weight). Control mice were treated with 200 µl PBS. Mice were sacrificed at day 7 or day 14 after instillation for collection of ATII cells.

### Primary murine ATII cell isolation and culture

Primary murine (pm) ATII cell isolation was performed as previously described (Corti et al., 1996; Königshoff et al., 2009). In brief, lungs of 8- to 10-week-old, pathogen-free female C57BL/6N mice (Charles River Laboratories, Sulzfeld, Germany) were lavaged with 500 µl of PBS twice. Lungs were flushed through the right heart using 0.9% NaCl solution (B. Braun Melsungen AG, Melsungen, Germany), inflated with 1.5 ml dispase (BD Bioscience, San Jose, CA) followed by 300 µl 1% low-melting-point agarose (Sigma-Aldrich, St Louis, MO) and incubated for 45 min at room temperature (RT). Subsequently, lungs were minced, filtered through 100 µm, 20 µm and 10 μm nylon meshes (Sefar, Heiden, Switzerland) and the cell suspension was centrifuged at 200 ***g*** for 10 min. The cell pellet was resuspended in DMEM cell culture medium (Sigma-Aldrich) and negative selection for macrophages and lymphocytes was performed by incubation of the single cell suspension on Petri dishes coated with antibodies against CD45 and CD16/32 (both BD Biosciences) for 30 min at 37°C. Non-adherent cells were collected and negative selection for fibroblasts was performed by adherence for 25 min on cell culture dishes. Cells were collected and cell viability was assessed by trypan blue exclusion. Cell purity was assessed by immunofluorescence staining of cells cultured overnight on chamber slides for proSFTPC, panCK, T1α, CD45, CD31 and αSMA. pmATII cells were resuspended in DMEM containing 10% FCS (PAA Laboratories, Pasching, Austria), 2 mM l-glutamine, 1% penicillin/streptomycin (both Life Technologies, Carlsbad, CA), 3.6 mg/ml glucose (Applichem GmbH, Darmstadt, Germany) and 10 mM HEPES (PAA Laboratories), and cultured for 24 h to allow attachment. Medium was changed and cells were cultured up to 5 days in a humidified atmosphere of 5% CO_2_ at 37°C with a medium change every other day.

### Cell culture treatments

PKF115-584-mediated inhibition of β-catenin activity in pmATII cells was performed by treatment with a concentration of 1 µM beginning at day 1 of culture until day 3 and day 5, respectively. Control cells were treated with the corresponding concentration of DMSO. Treatment and control media were refreshed at day 3. ICG-001 treatment (7.5 µM) was applied in the same manner. CT99021 treatment (2 µM) of cells was performed from day 1 to day 3. DMSO-treated cells served as control. The pharmacological inhibitors were purchased from the following companies: PKF115-584 (Santa Cruz), ICG-001 (Biomol) CT99021 (Tocris). siRNA-mediated downregulation of *Ctnnb1*, *Eno1* or *Pdia3* (ERp57) was performed using an siRNA pool of three target-specific siRNAs (*Ctnnb1* siRNA, *α-Enolase* siRNA, *ERp57* siRNA, Santa Cruz Biotechnology, Dallas, TX). Cells were transfected at day 2 after isolation using Lipofectamine RNAiMAX Transfection Reagent (Life Technologies) according to the manufacturer's instructions. Cells transfected with a scrambled control siRNA (siScr) (Santa Cruz Biotechnology, Dallas, TX) served as control. Non-transfected cells served as additional control. Cells were analyzed at day 5. siRNA efficiency was confirmed on mRNA level (data not shown) and protein level (Figs 4C and 6A,B).

### Cell viability assay

Cell viability analysis following ENO1 or PDIA3 siRNA-mediated knockdown was performed using the WST-1 assay (Roche, Basel, Switzerland) according to the manufacturer's instructions. The assay was performed at day 3 and day 5 and measured in triplicates.

### Immunofluorescence staining

Immunofluorescence staining was performed either on pmATII cells cultured overnight on chamber slides (BD Biosciences) for purity control or on cells cultured for 48 h on poly-l-lysine-coated coverslips for epithelial characterization. Cells were fixed with acetone/methanol (1:1), and blocked with 5% (w/vol) bovine serum albumin (BSA; Sigma-Aldrich) for 30 min. Cells were subsequently incubated with the respective primary antibody at RT for 1 h in PBS containing 0.1% (w/vol) BSA, followed by incubation with the fluorescently labeled secondary antibody (goat anti-rabbit Alexa Fluor 555, Life Technologies). DAPI staining (Roche) was used to visualize cell nuclei. Primary antibodies applied are the following: proSFTPC (Merck Millipore, Darmstadt, Germany), OCLN (Life Technologies), T1α (R&D Systems), PDIA3 (Abcam, Cambridge, UK).

### Quantitative (q)RT-PCR

Total RNA from pmATII was extracted using the QIAGEN RNeasy Mini Kit (Qiagen, Hilden, Germany) according to the manufacturer's instructions. cDNA of all samples was generated by reverse transcription using SuperScriptII (Life Technologies). Quantitative real-time PCR (qRT-PCR) was performed using SYBR Green and the LightCycler 480 System (both Roche). Hypoxanthine guanine phosphoribosyl transferase (*Hprt*) was utilized as a reference gene in all qRT-PCR reactions. The following primers were used in a final concentration of 200 nM: *Ager* (NM_007425.3; NM_001271422.1; NM_001271423.1), fw 5′-CTCGAATCCTCCCCAATGGT-3′, rv 5′-CCAGGAATCTGGTAGACTCGG; *Axin2* (NM_015732.4), fw 5′-AGCAGAGGGACAGGAACCA-3′, rv 5′-CACTTGCCAGTTTCTTTGGCT-3′; *Cbr2* (NM_007621.2), fw 5′-AGGAAGTTCGCAGAGGTTGA-3′, rv 5′-GGCAACTGAGCAGACTAGGA-3′; *Cdh1* (NM_009864.2), fw 5′-CCATCCTCGGAATCCTTGG-3′, rv 5′-TTTGACCACCGTTCTCCTCC-3′; *Dkk2* (NM_020265.4), fw 5′-GAGATCGCAACCATGGTCACT-3′, rv 5′-GGGTCTCCTTCATGTCCTTTTATATG-3′; *Eno1* (NM_023119.2), fw 5′-ACCCTCTTTCCTTGCTTTGC-3′, rv 5′-GAAGAGACCTTTTGCGGTGT-3′; *FoxM1* (NM_008021.4), fw 5′-ACGCTGGACAACAGCTTAAC-3′, rv 5′-AGGGCTCCTCAACCTTAACC-3′; *Hprt* (NM_013556.2), fw 5′-CCTAAGATGAGCGCAAGTTGAA-3′, rv 5′-CCACAGGACTAGAACACCTGCTAA-3′; *Pdia3* (NM_007952.2), fw 5′-AGCAAAGGTGGATTGCACTG-3′, rv 5′-CCATCATAAGCACCCGCTTC-3′; *Sftpa* (NM_023134.4), fw 5′-GGAGAGCCTGGAGAAAGGGGGC-3′, rv 5′-ATCCTTGCAAGCTGAGGACTCCC-3′; *Sftpc* (NM_011359.2), fw 5′-AGCAAAGAGGTCCTGATGGA-3′, rv 5′-GAGCAGAGCCCCTACAATCA-3′; *T1α* (NM_010329.2), fw 5′-ACAGGTGCTACTGGAGGGCTT-3′, rv 5′-TCCTCTAAGGGAGGCTTCGTC-3′; *Tjp1* (NM_009386.2; NM_001163574.1), fw 5′-ACGAGATGCTGGGACTGACC-3′, rv 5′-AACCGCATTTGGCGTTACAT-3′; *Wnt3a* (NM_009522.2), fw 5′-GCACCACCGTCAGCAACA-3′, rv 5′-GGGTGGCTTTGTCCAGAACA-3′; *Wnt10a* (NM_009518.2), fw 5′-GCCCCATCTTCAGCCGAGGTTT-3′, rv 5′-CGTCGCAACCGCAAGCCTTC-3′; *Wnt10b* (NM_011718.2), fw 5′-TGGGACGCCAGGTGGTAA-3′, rv 5′-CTGACGTTCCATGGCATTTG-3′. Relative transcript levels are expressed in ΔCt values (ΔCt=Ct^reference^−Ct^target^) or log-fold change (ΔΔCt values).

### Immunoblotting

Cells were washed twice with PBS (PAA Laboratories), lysed in T-PER lysis buffer (Thermo Fisher Scientific, Waltham, MA) supplemented with proteinase inhibitor cocktail tablets (Roche), and lysates were centrifuged at 5660 ***g*** at 4°C. Supernatant was collected and protein concentration was determined using the Quick Start Bradford Dye Reagent according to the manufacturer's instructions. 15 µg of total protein was separated on SDS-polyacrylamide gels and transferred to nitrocellulose membranes (Bio-Rad, Hercules, CA). Membranes were blocked in 5% nonfat dry milk (Applichem) or 5% BSA (Sigma-Aldrich) in TRIS-buffered saline containing 0.05% (v/v) Tween (TBST) (Applichem) and incubated with the primary antibody at 4°C overnight. The respective HRP-labeled secondary antibody (anti-rabbit-HRP antibody and anti-mouse-HRP antibody, both GE Healthcare, Chalfont St Giles, UK; anti-goat-HRP, Life Technologies) was applied after washing of the membrane in TBST. Proteins were visualized using the SuperSignal West Dura Chemiluminescent Substrate (Thermo Fisher Scientific) and the ChemiDoc™ XRS+ system (Bio-Rad). The following primary antibodies were used: proSFTPC (Abcam), T1α (R&D Systems), active β-catenin (ABC; Merck Millipore), CBR2 (Avida Systems Biology, San Diego, CA), PDIA3 (Santa Cruz Biotechnology), ENO1 (Cell Signaling), DVL3 (Santa Cruz Biotechnology) and TJP1 (Life Technologies). β-actin served as loading control and was detected using a HRP-conjugated anti-β-actin antibody (Sigma-Aldrich). Densitometric analysis of band intensities was performed using Image Lab 5.0 software from Bio-Rad.

### Protein extraction for two-dimensional gel electrophoresis

Primary ATII cells were washed twice with PBS (PAA Laboratories) and lysed in rehydration buffer (9 M urea, 4% CHAPS, 100 mM DTT) at RT. The samples were subsequently centrifuged at 5660 ***g*** for 30 min at 20°C. After centrifugation, the supernatant was collected and the protein concentrations were determined by Quick Start Bradford Dye Reagent using a SmartSpec 3000 spectrophotometer (both Bio-Rad Laboratories).

### Two-dimensional electrophoresis

Isoelectric focusing (IEF) was carried out using the PROTEAN IEF Cell and immobilized pH gradient (IPG) strips (7 cm; pH 3-10; both from Bio-Rad Laboratories). IPG strips were rehydrated overnight at 50 V with 0.2% ampholytes and pH 3-10 (Bio-Rad Laboratories) together with 20 µg of sample for day 1, day 3 and day 5. IEF was performed under the following conditions: 100 V, 1 h; 250 V, 1 h; 750 V, 1 h; 1000 V, 1 h; 2500 V, 1 h; 12 kV, 1 h. The strips were maintained at −80°C until further use. Prior to second dimension, the strips were equilibrated for 15 min in 10 ml equilibration buffer [6 M urea, 0.375 M Tris-HCl (pH 8.8), 2% (v/v) SDS, 20% (v/v) glycerol] containing 2% (w/v) DTT and subsequently for 15 min in equilibration buffer containing 2.5% (w/v) iodoacetamide. The separation in the second dimension was realized on SDS polyacrylamide gels (12.5%).

### 2D-gel analysis

The gels were individually stained with SyproRuby fluorescent stain (Bio-Rad Laboratories) according to the manufacturer's instructions. All gels were scanned at 100 mm resolution using the Molecular Imager FX™ at an excitation wavelength of 532 nm and an emission wavelength filter of 610 nm. Images produced from three independent extracts for each time point were converted into digital TIF files. Spot detection, pattern evaluation and normalization were performed using the PDQuest 2-D Analysis Software (version 7.2, Bio-Rad Laboratories). One gel from day 3 cell culture was set as master gel. Protein spots were automatically detected and visually checked for undetected or incorrectly detected spots and then matched to their corresponding spots in a digitized master gel. Intensity levels were normalized between gels by the total quantity in valid spots of gel images. In order to excise proteins or polypeptides from the gels they were visualized by silver staining.

### Tryptic digestion and MALDI-TOF MS

Proteins were identified using Pick'n’Post Protein identification service (Alphalyse, Odense, Denmark). Briefly, gel-excised protein spots were reduced, alkylated with iodoacetamide, and digested with trypsin. The resulting peptides were concentrated on a Zip-Tip C18 column (Merk Millipore) and eluted onto an anchorchip target for analysis on a Bruker Autoflex III MALDI TOF/TOF instrument (Billerica, MA). The peptide mixture was analyzed in positive reflector mode for accurate peptide mass determination and some of the peptides analyzed by MS/MS fragmentation for partial-peptide sequencing. For acquisition of peptide mass fingerprint spectra (PMF, MS), 3000 single-shot spectra were averaged and the peak finding was undertaken using the SNAP algorithm. Peptide fragmentation spectra (PFF, MS/MS) were acquired when possible. The MS and MS/MS spectra were combined and used for a MASCOT database search (MASCOT version 2.1.03) in the NCBI protein database. For PFF spectra, the mass tolerance was set at 60 ppm, allowing one missed cleavage site.

### Statistical analysis

Results are presented as mean+s.e.m. and were considered statistically significant when *P*<0.05. Means of respective groups were compared using a one-way ANOVA, followed by the Dunnett's post-hoc test or Bonferroni multiple-comparison test or two-tailed *t*-test as indicated in the figure legends. Linear regression analysis was used to determine correlation of mRNA expression of different genes. All statistical analysis was performed using GraphPad Prism5.

## Supplementary Material

Supplementary Material
