# Supplementary material for: Enolase 1 (ENO1) and protein disulfide-isomerase associated 3 (PDIA3) regulate Wnt/β-catenin-driven trans-differentiation of murine alveolar epithelial cells
Source: Dis Model Mech. 2015 Aug 1;8(8):877–90. doi: 10.1242/dmm.019117 (PMC4527283; doi:10.1242/dmm.019117)
Supplement: Supplementary Material [file supp_8_8_877__index.html]

Supplementary Material 

# Enolase 1 and protein disulfide isomerase associated 3 regulate Wnt/β-catenin driven alveolar epithelial cell trans-differentiation

## DMM019117 Supplementary Material

- Supplementary Material
