# Supplementary material for: Enolase 1 (ENO1) and protein disulfide-isomerase associated 3 (PDIA3) regulate Wnt/β-catenin-driven trans-differentiation of murine alveolar epithelial cells
Source: Dis Model Mech. 2015 Aug 1;8(8):877–90. doi: 10.1242/dmm.019117 (PMC4527283; doi:10.1242/dmm.019117)
Supplement: Supplementary Material [file supp_019117_DMM019117supp.pdf]

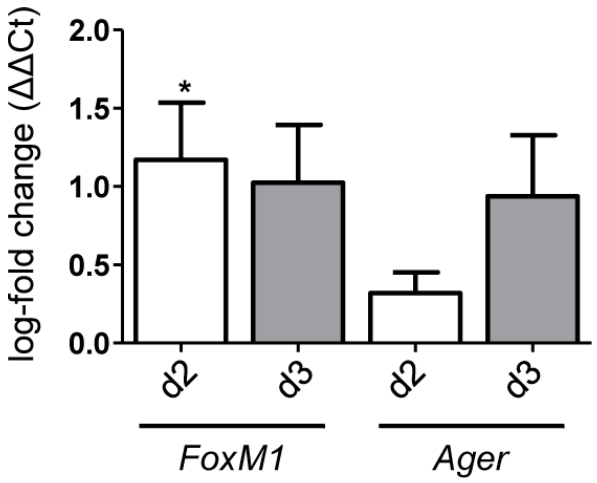

Fig. S1. Expression of *FoxM1* and *Ager* in pmATII cells. mRNA expressions of *FoxM1* and *Ager* during culture of pmATII cells. mRNA levels were measured by quantitative RT-PCR (qRT-PCR) and normalized to *Hprt* as housekeeping gene. Data represent means of ΔΔCt values (log-fold change) + s.e.m. of at least 3 independent experiments. Means at indicated time points were compared to day 1 using one-way ANOVA, followed by Dunnett's post hoc test. Significance: * $P<0.05$

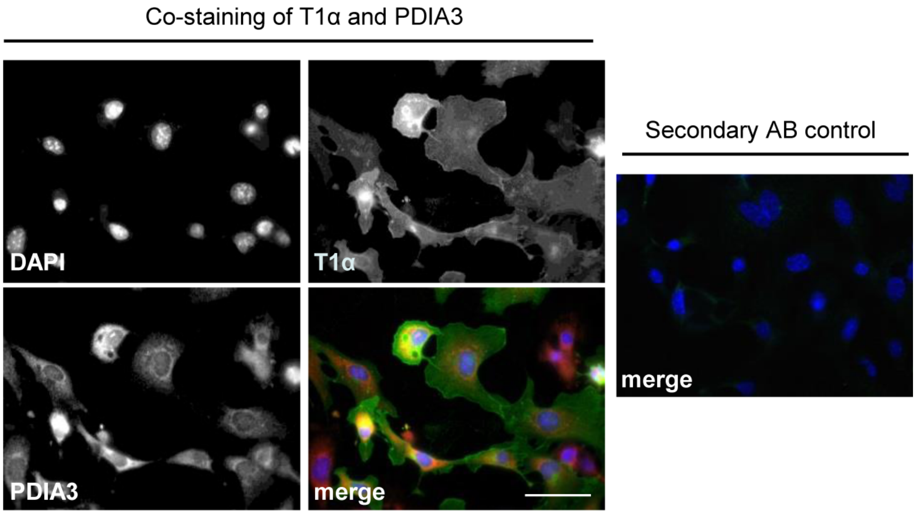

Fig. S2. Co-expression of T1α and PDIA3 in pmATII cells. Immunofluorescence staining of pmATII cells on cover slips for T1α and PDIA3 at day 3 after isolation. Fluorescent images represent a 400 x magnification. The scale bar represents 50 µm. A merged image of the secondary antibody control is shown.

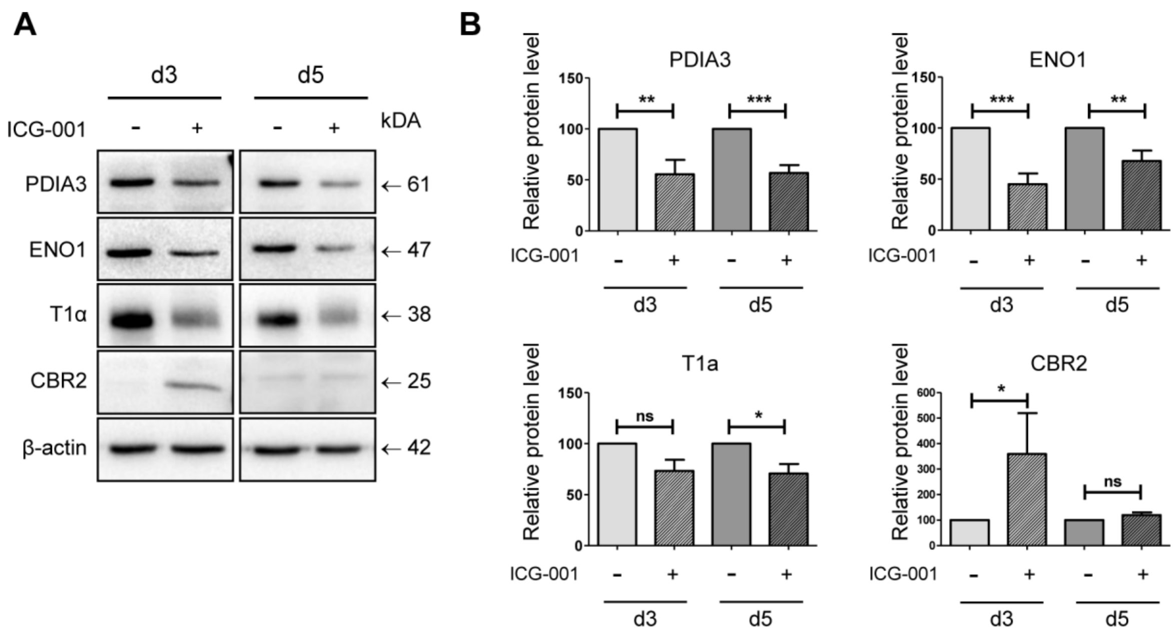

Fig. S3. β-catenin inhibition using ICG-001 alters ATII to ATI cell trans-differentiation along with CBR2, ENO1 and PDIA3 expression. (A) pmATII were treated with ICG-001 or DMSO as control at d1 after isolation until day 3 and day 5, respectively. Treated cells were lysed and subjected to immunoblot analysis. β-actin expression served as loading control. (A) A representative experiment is shown. (B) Densitometric analysis of at least 3 independent experiments using ICG-001 treatment. Means of the indicated groups were compared to time matched treatment controls using one-way ANOVA, followed by Bonferroni multiple comparison test. Significance: * P<0.05; ** P<0.01; *** P<0.001. ns not significant.

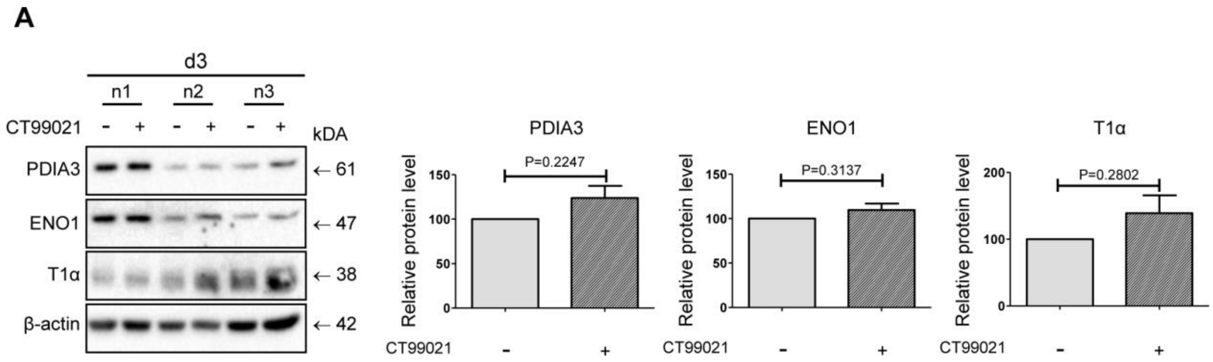

Fig. S4. Treatment of pmATII cells with β-catenin activator CT99021 (A) pmATII were treated with CT99021 (2µM), or DMSO as control at d1 after isolation until day 3. Treated cells were lysed and subjected to immunoblot analysis. β-actin expression served as loading control. (A) 3 independent experiments are shown. (B) Densitometric analysis of 3 independent experiments using CT99021 treatment. Means of the indicated groups were compared using a two-tailed, paired t-test.

SUPPLEMENTARY TABLE S1

Proteins identified on 2D gel by MALDI-TOF MS

Number	Name of protein	GI-number	Unique peptides detected	Mascot score	Sequence coverage	Peptide sequences used for MS/MS
**1**	Calreticulin	6680836	15	181	30%	^{56}FYGDLEKDK^{64}<br>^{143}KVHVIFNYK^{151}<br>^{144}VHVIFNYK^{151}
**2**	Protein disulfide-isomerase	54777	19	318	33%	^{72}LKAEGSEIR^{80}<br>^{84}VDATEESDLAQQYGVR^{99}<br>^{211}FDEGRNNFEGEITK^{224}<br>^{447}FFPASADR^{454}
**3**	Carbonyl reductase 2	6671688	12	123	34%	^{1}MKLNFSGLR^{9}<br>^{110}SVFQVSQMVAR^{120}<br>^{190}VSADPEFAR^{198}
**4**	ATP synthase subunit β, mitochondrial	74197074	21	337	42%	^{144}IMNVIGEPIDER^{155}<br>^{265}VALVYGQMNEPPGAR^{279}<br>^{311}FTQAGSEVSALLGR^{324}<br>^{388}AIAELGIYPAVDPLDSTSR^{406}
**5**	Hspd1 protein	76779273	17	219	31%	^{97}LVQDVANNTNEEAGDGTTTATVLAR^{121}<br>^{134}GANPVEIRR^{142}<br>^{430}AAVEEGIVLGGGCALLR^{446}<br>^{463}IGIEIIKR^{470}
**6**	Putative uncharacterized protein	74191337	10	249	29%	^{133}LYGPSSVSFADDFVR^{147}<br>^{393}DNQSGSLLFIGR^{404}
**7**	Protein disulfide isomerase associated 3	23958822	23	339	36%	^{108}DGEEAGAYDGPR^{119}<br>^{148}FISDKDASVVGFFR^{161}<br>^{472}ELNDFISYLQR^{482}
**8**	Krt8 protein	76779293	26	346	48%	^{84}LEVDPNIQAVR^{94}<br>^{140}SNMDNMFESYINNLR^{154}<br>^{220}LEGLTDEINFLR^{231}
**9**	Anxa1	70912321	17	444	44%	^{114}TPAQFDADELR^{124}<br>^{129}GLGTDEDTLIEILTTR^{144}<br>^{214}KGTDVNVFTTILTSR^{228}<br>^{215}GTDVNVFTTILTSR^{228}
**10**	Caldesmon 1	18043856	24	357	40%	^{104}RLQEALER^{111}<br>^{112}QKEFDPTITDGSLSGPSR^{129}<br>^{151}SGRYEVEETEVVIK^{154}<br>^{242}QTENAFSPSR^{251}
**11**	Enolase 1	54673814	12	277	34%	^{33}AAVPSGASTGIYEALELR^{50}<br>^{163}LAMQEFMILPVGASSFR 2^{179}<br>^{257}YDLDFKSPDDPSR^{269}<br>^{270}YITPDQLADLYK^{281}
**12**	Aldehyde dehydrogenase 2	6753036	19	363	38%	^{110}LADLIERDR^{118}<br>^{162}TIPIDGDFFSYTR^{174}<br>^{327}TFVQENVYDEFVER^{340}<br>^{349}VVGNPFDSR^{357}<br>^{431}TIEEVVGR^{438}
**13**	Serine hydroxymethyl transferase 2	21312298	22	338	39%	^{70}GLELIASENFCSR^{82}<br>^{96}YSEGYPGKR^{104}<br>^{220}LIIAGTSAYAR^{230}<br>^{357}NAQAMADALLKR^{368}<br>^{426}LGAPALTSR^{434}

						^{470}SFLLKDPETSQR^{481}
**14**	ATP synthase,	6680748	19	327	37%	^{59}ILGADTSVDLEETGR^{73}<br>^{134}TGAIVDVPVGEELLGR^{149}<br>^{195}AVDSLVPIGR^{204}<br>^{335}EAYPGDVFYLHSR^{441}
**15/16**	Lamin A/C	1794160	44	728	55%	^{12}SGAQASSTPLSPTR^{25}<br>^{29}LQEKEDLQELNDR^{41}<br>^{51}SLETENAGLR^{60}<br>^{281}NSNLVGAAHEELQQSR^{296}<br>^{320}IRIDSLSAQLSQLQK^{329}<br>^{379}LLEGEEERLR^{388}<br>^{440}VAVEEVDEEGKFVR^{453}
**17**	Mitochondrial phosphoenolpyruvate carboxykinase 2	28077029	22	225	35%	^{145}DTVPLLAGGAR^{155}<br>^{262}TLIGHVPDQR^{271}<br>^{262}FDSEGQLR^{269}<br>^{497}HGVFVGSAMR^{506}<br>^{588}RLEGEDSAQETPIGLVPK^{605}<br>^{628}DFWEQEVR^{635}
**18**	Mitochondrial phosphoenolpyruvate carboxykinase 2	28077029	21	228	35%	^{145}DTVPLLAGGAR^{155}<br>^{262}TLIGHVPDQR^{271}<br>^{362}FDSEGQLR^{369}<br>^{496}HGVFVGSAMR^{506}<br>^{588}RLEGEDSAQETPIGLVPK^{605}<br>^{628}DFWEQEVR^{635}
**19**	Dihydrolipoyl dehydrogenase,	6014973	12	154	23%	^{133}ALTGGIAHLFK^{143}<br>^{335}GRIPVNNR^{342}<br>^{418}IGKFPFAANSR^{428}<br>^{483}VCHAHPTLSEAFR^{495}
**20**	Glutamate dehydrogenase 1	6680027	8	150	16%	^{69}MVEGFFDR^{76}<br>^{125}DDGSWEVIEGYR^{136}<br>^{481}HGGTIPVVPTAEFQDR^{496}
**21**	Annexin A11	7304885	18	112	33%	^{358}DVQELYAAGENR^{369}<br>^{387}AHLVAVFNEYQR^{398}
**22**	Heat shock 70kD protein 5	29748016	27	548	36%	^{62}ITPSYVAFTPEGER^{75}<br>^{166}VTHAVVTVPAYFNDAQR^{182}<br>^{199}IINEPTAAAIAYGLDKR^{215}<br>^{326}AKFEELNMDLFR^{337}<br>^{354}KSDIDEIVLVGGSTR^{368}
**23**	Electron transferring flavoprotein,	38142460	13	241	49%	^{60}EIIAVSCGPSQCQETIR^{76}<br>^{165}EIDGGLETLR^{174}<br>^{175}LKLPAVVTADLR^{186}<br>^{222}VSVISVEEPPQR^{233}
**24**	Malate dehydrogenase 2	31982186	6	203	21%	^{92}GCDVVVIPAGVPR^{104}<br>^{166}IFGVTTLDIVR^{176}<br>^{216}VDFPQDQLATLTGR^{229}
**25**	Heterogeneous nuclear ribonucleoprotein A2/B1/B0	23266713	8	155	28%	^{138}IDTIEIITDR^{147}<br>^{204}GGNFGFGDSR^{213}<br>^{214}GGGGNFGPGPGSNFR^{228}
**26**	Caldesmon 1	18043856	25	233	43%	^{112}QKEFDPTITDGSLSGPSR^{129}<br>^{242}QTENAFSPSR^{251}<br>^{342}LKEEIER^{348}
**27**	Ornithine aminotransferase	8393866	9	237	21%	^{32}KTEQGPPSSEYIFER^{46}<br>^{33}TEQGPPSSEYIFER^{46}<br>^{414}LAPPLVIKEDEIR^{426}
